# Coronavirus disease 2019 and stroke in Iran: a case series and effects on stroke admissions

**DOI:** 10.1177/1747493020937397

**Published:** 2020-06-26

**Authors:** M Mehrpour, A Shuaib, M Farahani, HR Hatamabadi, Z Fatehi, M Ghaffari, NB Moghadam, SH Aghamiri, B Mansouri, F Assarzadegan, BS Lima, O Hesami

**Affiliations:** 1 Department of Neurology, Shahid Beheshti University of Medical Sciences, Tehran, Iran; 2 Department of Medicine, University of Alberta, Edmonton, Canada; 3 Iranian Stroke Organization, Tehran, Iran; 4 Department of Emergency Medicine, Shahid Beheshti University of Medical Sciences, Tehran, Iran

**Keywords:** Coronavirus disease 2019, pandemic, neurology, cerebrovascular disease, stroke, acute stroke

## Abstract

**Objective:**

The coronavirus disease 2019 pandemic has affected healthcare systems around the globe and massively impacted patients with various non-infectious, life-threatening conditions. Stroke is a major neurological disease contributing to death and disability worldwide, and is still an ongoing issue during the pandemic. Here we investigate the impact of the coronavirus disease 2019 outbreak on stroke manifestations, treatment courses, the outcome of stroke patients, and the hospitalization rate in a referral center for stroke management in Tehran, Iran.

**Methods:**

We extracted data regarding 31 stroke patients (10 patients with laboratory-confirmed coronavirus disease 2019) and compared the demographic and pathological characteristics of the patients with or without coronavirus disease 2019 infection. The association of demographic/pathological characteristics of stroke patients during the coronavirus disease 2019 pandemic and a corresponding period during the previous year (49 patients) and an earlier period during the same year as the pandemic (50 patients) was also evaluated.

**Results:**

The absolute number of admissions decreased about 40% during the coronavirus disease 2019 pandemic. Except for the stroke severity (P = 0.002), there were no significant changes in the demographic and pathological characteristics of the stroke patients during the three studied periods. A significantly higher mean of age (75.60 ± 9.54 versus 60.86 ± 18.45; P = 0.007), a significant difference in the type of stroke (P = 0.046), and significantly higher stroke severity (P = 0.024) were observed in stroke patients with coronavirus disease 2019 compared with those of stroke patients without coronavirus disease 2019. Treatment approaches, duration of hospitalization, and mortality rates did not differ significantly.

**Conclusions:**

This report shows that the pandemic caused the number of acute stroke admissions to plummet compared to other periods. Although the pandemic did not affect the treatment plans and care of the patients, stroke cases with coronavirus disease 2019 had higher age, more large vessel ischemic stroke, and more severe stroke. Further studies are urgently needed to realize the probable interaction of the coronavirus disease 2019 pandemic and the neurologic disease.

## Introduction

As the world grapples with coronavirus disease 2019 (COVID-19), healthcare systems globally are adversely affected and interference with the care-seeking, diagnosis, and treatment of non-infectious disease has been reported.^1^ The fear of coronavirus has resulted in a decrease in the number of acute stroke admissions all around the world, raising alarm that effective treatment may be delayed or denied.^2^

Stroke is one of the main causes of death and functional disability worldwide. More than 100,000 people in Iran suffer from stroke annually.^3^

**Figure 1.**
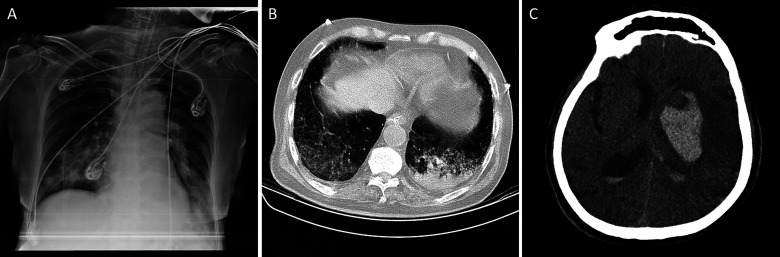
Chest X-ray (a) and chest computer-assisted tomography scan without contrast of chest (b) and brain (c) of a COVID-19 positive patient with severe hemorrhagic stroke.

**Figure 2.**
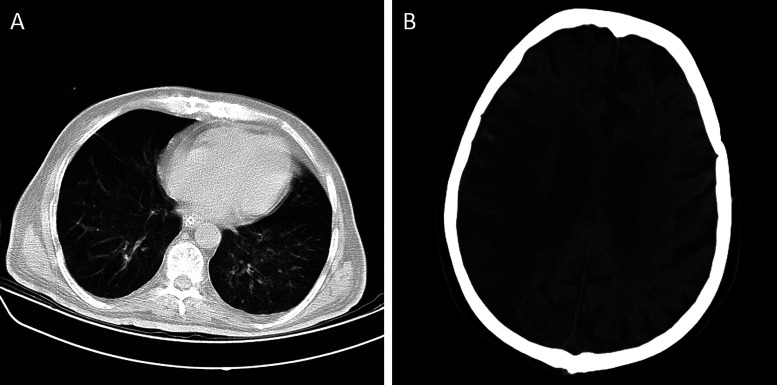
Computer-assisted tomography scan of chest (a) and brain (b) without contrast in a COVID-19 positive patient with severe large vessel ischemic stroke.

Iran had a high number of COVID-19 patients early in the pandemic. Accordingly, to explore the hospitalization rate, treatment course, and outcome of stroke patients in the stroke unit during the COVID-19 pandemic and their differences in comparison to the usual, we performed a retrospective clinical analysis of patients admitted for stroke at our center, in three two-month-long periods.

## Materials and methods

### Ethical statement

The study was approved by the institutional review board of the Iran Stroke Organization, and written informed consent was obtained from all participants.

### Patients

All the patients’ data were retrospectively retrieved from the medical records of the hospitalized patients in Imam Hossein Hospital, the main referral center for stroke management in the east of Tehran, Iran. All information related to stroke cases, admitted during three periods of 15 February 2019 to 15 April 2019, 15 September 2019 to 15 February 2020 (before the identification of patient-zero of COVID-19 in Iran), and 15 February 2020 to 15 April 2020 (during the pandemic) were gathered and classified as last year, before COVID-19, and during COVID-19 groups, respectively.

The severity of the condition for each case was determined according to the patients’ National Institutes of Health Stroke Scale (NIHSS) score.^4^ All patients with NIHSS score more than 20 were considered severe, and the others were deemed non-severe. COVID-19 positive cases were diagnosed with quantitative reverse-transcriptase polymerase chain reaction test of nasal or pharyngeal swab samples and were confirmed with high-resolution computer-assisted tomography of the lungs. In cases that CAT-scan was negative for COVID-19, but either of the nasal or pharyngeal swab samples was positive, confirmation was performed by a repeat nasal or pharyngeal swab test. The degree of severity of COVID-19 (mild, moderate, severe, critical) was defined using the WHO-China Joint Mission on Coronavirus Disease 2019 (COVID-19) report.^5^

### Statistical analysis

All statistical analyses were performed using SPSS 20.0 (SPSS, Inc., Chicago, IL, USA). Quantitative variables (age, duration of hospitalization) were presented as the mean ± standard deviation, and the one-way analysis of variance test, the independent t-test, or the Mann–Whitney U test was applied to compare differences between groups. Categorical variables were presented as percentages and determined by using the chi-square (χ2) test. P < 0.05 was considered to indicate a statistically significant difference.

## Results

During the COVID-19 pandemic, we had fewer patients (31) in comparison to period of the same duration immediately prior to patient-0 in Iran (49) and over the similar period the previous year (50). The decrease in the number of admissions during the COVID-19 pandemic was approximately 40% compared to the other two periods.

The mean of age was similar in all three periods (p-value = 0.665): 65.61 during the COVID-19 period, 64.24 for the period before, and 67 during last year. There was a higher proportion of severe strokes admitted during the COVID-19 period (Table 1). The proportion of large vessel ischemic stroke during the pandemic period was 38.7%, compared with 26.5 and 14% during the immediate period preceding and the previous year, respectively. Non-large vessel ischemic stroke during the pandemic accounted for 35.5% of all patients, compared to 49% immediately before the pandemic and 64% the previous year. Hemorrhagic stroke during all three periods was similar, with 25.8% during the epidemic, 24.5% immediately before the pandemic, and 22% the previous year.

**Table 1.** Clinical characteristics of stroke patients during the COVOID-19 pandemic, in the immediately preceding period and during a similar period of the previous year.

Time period	During COVID-19	Immediately Before COVID-19	Previous year	P-value
Number	31	49	50	
Sex				0.358
Male	20 (64.5%)	30 (61.2%)	25 (50.0%)	
Female	11 (35.5%)	19 (38.8%)	25 (50.0%)	
Age (year)^a^	65.61 ± 17.41	64.24 ± 13.99	67.00 ± 14.77	0.665
Type of stroke				0.088
Hemorrhagic stroke	8 (25.8%)	12 (24.5%)	11 (22.0%)	
Non-large vessel ischemic	11 (35.5%)	24 (49.0%)	32 (64.0%)	
Large vessel ischemic	12 (38.7%)	13 (26.5%)	7 (14.0%)	
Treatment				0.055
Medical treatment	22 (71.0%)	34 (69.4%)	34 (68.0%)	
Thrombolytic (TPA)	4 (12.9%)	8 (16.3%)	14 (28.0%)	
Thrombectomy	3 (9.7%)	6 (12.2%)	0	
Thrombectomy and TPA	2 (6.5%)	1 (2.0%)	0	
Non-medical	0	0	2 (4.0%)	
Severity of stroke^b^				0.002*
Non-severe	12 (38.7%)	20 (40.8%)	36 (72.0%)	
Severe	19 (61.3%)	29 (59.2%)	14 (28.0%)	
Duration of hospitalization (day)^a^				
Patients with non-severe stroke	1.75 ± 0.97	2.30 ± 1.87	3.00 ± 1.96	0.090
Patients with severe stroke	6.63 ± 4.67	6.45 ± 4.15	6.93 ± 4.57	0.945
Mortality	5 (16.1%)	10 (20.4%)	7 (14.0%)	0.690

COVID-19: coronavirus disease 2019.

* Significant difference (p < 0.05).

a Mean ± SD

b The method of classification for stroke severity is explained in the text.

The percentage of thrombolysis and thrombectomy treatments was similar during and before the COVID-19 period (P-value = 0.055).

Table 2 shows the statistical analysis of stroke patients with or without COVID-19 infection. COVID-19 positive stroke cases were older (75.60 ± 9.54 versus 60.86 ± 18.45), had a higher percentage of large vessel ischemic stroke (70% versus 23.8%), and a high proportion had severe stroke (90.0% versus 47.6%).

**Table 2.** Comparison of clinical characteristics of stroke patients with and without COVID-19 infection.

	During COVID-19 pandemic		P-value
Parameter	Negative COVID-19 infection	Positive COVID-19 infection	
Number	21	10	
Sex			0.244
Male	15 (71.4%)	5 (50.0%)	
Female	6 (28.6%)	5 (50.0%)	
Age (year)^a^	60.86 ± 18.45	75.60 ± 9.54	0.007*
Type of stroke			0.046*
Hemorrhagic stroke	7 (33.3%)	1 (10.0%)	
Non-large vessel ischemic	9 (42.9%)	2 (20.0%)	
Large vessel ischemic	5 (23.8%)	7 (70.0%)	
Treatment			0.333
Medical treatment	17 (81.0%)	5 (50.0%)	
Thrombolytic (TPA)	2 (9.5%)	2 (20.0%)	
Thrombectomy	1 (4.8%)	2 (20.0%)	
Thrombectomy and TPA	1 (4.8%)	1 (10.0%)	
Severity of stroke^b^			0.024*
Non-severe	11 (52.4%)	1 (10.0%)	
Severe	10 (47.6%)	9 (90.0%)	
Severity of COVID-19 infection^c^			
Mild	–	4 (40.0%)	
Moderate	–	2 (20.0%)	
Severe	–	3 (30.0%)	
Critical	–	1 (10.0%)	
Duration of hospitalization (day)^a^			
Patients with non-severe stroke	1.55 ± 0.69	4.00	0.083
Patients with severe stroke	6.30 ± 5.17	7.00 ± 4.33	0.754
Mortality	2 (9.5%)	3 (30.0%)	0.147

COVID-19: coronavirus disease 2019.

* Significant difference (p < 0.05).

a Mean ± SD.

b The method for classification of stroke severity is explained in the text.

c The method for classification of COVID-19 severity is explained in the text.

## Discussion

Recent evidence suggests that there is a high incidence of neurological complications, including cerebrovascular disease in patients diagnosed with COVID-19.^6^ In this report, we analyzed admission data during the coronavirus pandemic and compared this to stroke admission from a similar duration period immediately before the pandemic and period from the previous year.

The most important difference was a reduction in the number of patients admitted to the hospital with the diagnosis of stroke during the pandemic, as previously reported in other countries.^6-8^ A similar reduction has been reported in admission with acute coronary syndrome in both Italy and the USA^9,10^ suggesting that people with different life-threatening diseases are refraining from approaching hospitals due to fear of exposure to the virus. Stroke has been reported to worsen the prognosis of COVID-19^11^ resulting in the recommendation that elderly patients with stroke and other cardiovascular comorbidities avoid contact with other people and high possible risk environments, which could include hospitals.

Our results demonstrated an increase in large vessel ischemic stroke both during the pandemic period compared with other time periods, among stroke patients presenting with COVID-19 compared with COVID-19 negative patients during the same period. This is consistent with a previous report from a small case series^12^; unlike the report results of that study, the average age among COVID-19 cases in our series with large vessel ischemic stroke was higher. All stroke cases were older than 60 years of age with a mean age of 75.60 ± 9.54 years old.

A major finding of this study was that stroke severity was significantly affected by the COVID-19 infection. Significantly higher stroke severity was observed in stroke patients with COVID-19 compared with those of stroke patients without COVID-19. The treatment of stroke did not appear to differ during the pandemic, and thrombolysis and thrombectomy rates remained similar.

There are limitations to our study. First, we had a relatively small sample size, and the study is not a true representation of the general population in Iran. Large multicenter studies are required to better understand the relationship of COVID-19 to stroke. We are in preparation for a collaborative study with data gathered from all major hospitals in Iran. Second, the study data were collected in a retrospective manner.

## Conclusion

Iran has been severely affected by the COVID-19 pandemic. Our study demonstrates a reduction in stroke admissions in Iran during the pandemic. We also described the characteristics of stroke in COVID-19 positive patients in Iran. COVID-19 was associated with an increase in large artery stroke and an increase in stroke severity.
